# MUTYH DNA glycosylase: the rationale for removing undamaged bases from the DNA

**DOI:** 10.3389/fgene.2013.00018

**Published:** 2013-02-28

**Authors:** Enni Markkanen, Julia Dorn, Ulrich Hübscher

**Affiliations:** Institute for Veterinary Biochemistry and Molecular Biology, University of Zürich-IrchelZürich, Switzerland

**Keywords:** MUTYH, MUTYH-associated polyposis (MAP), MYH, mutY, DNA polymerase beta and lambda, base-excision repair (BER), DNA glycosylases, 8-oxo-guanine

## Abstract

Maintenance of genetic stability is crucial for all organisms in order to avoid the onset of deleterious diseases such as cancer. One of the many proveniences of DNA base damage in mammalian cells is oxidative stress, arising from a variety of endogenous and exogenous sources, generating highly mutagenic oxidative DNA lesions. One of the best characterized oxidative DNA lesion is 7,8-dihydro-8-oxoguanine (8-oxo-G), which can give rise to base substitution mutations (also known as point mutations). This mutagenicity is due to the miscoding potential of 8-oxo-G that instructs most DNA polymerases (pols) to preferentially insert an Adenine (A) opposite 8-oxo-G instead of the appropriate Cytosine (C). If left unrepaired, such A:8-oxo-G mispairs can give rise to CG→AT transversion mutations. A:8-oxo-G mispairs are proficiently recognized by the MutY glycosylase homologue (MUTYH). MUTYH can remove the mispaired A from an A:8-oxo-G, giving way to the canonical base-excision repair (BER) that ultimately restores undamaged Guanine (G). The importance of this MUTYH-initiated pathway is illustrated by the fact that biallelic mutations in the *MUTYH* gene are associated with a hereditary colorectal cancer syndrome termed MUTYH-associated polyposis (MAP). In this review, we will focus on MUTYH, from its discovery to the most recent data regarding its cellular roles and interaction partners. We discuss the involvement of the MUTYH protein in the A:8-oxo-G BER pathway acting together with pol λ, the pol that can faithfully incorporate C opposite 8-oxo-G and thus bypass this lesion in a correct manner. We also outline the current knowledge about the regulation of MUTYH itself and the A:8-oxo-G repair pathway by posttranslational modifications (PTM). Finally, to achieve a clearer overview of the literature, we will briefly touch on the rather confusing MUTYH nomenclature. In short, MUTYH is a unique DNA glycosylase that catalyzes the excision of an undamaged base from DNA.

## Introduction

Cellular DNA is constantly under attack of damaging agents, such as reactive oxygen species (ROS), that derive from a multitude of exogenous and endogenous sources (reviewed in Van Loon et al., 2010). One of the main consequences of ROS impact on DNA is the formation of 8-oxo-G, a frequent DNA lesion estimated to arise around 1000–7000 times per cell per day (Collins, 1999; European Standards Committee on Oxidative DNA Damage (ESCODD), 2003; Gedik and Collins, 2005; Friedberg, 2006). To counteract this heavy burden of 8-oxo-G lesions, a multi-component system involving a plethora of enzymes has evolved both in bacteria and mammals. 8-oxo-dGTP, which arises upon oxidation of the nucleotide pool, is hydrolyzed by the enzymes MutT/MTH1, which therefore prevent incorporation of 8-oxo-dGTP into nascent DNA. When a C:G base pair is oxidized to C:8-oxo-G, the enzyme Fpg (also known as MutM)/OGG can catalyze the removal of 8-oxo-G from these base pairs. Furthermore, other proteins such as the mismatch-repair pathway component MutS/MSH2, or the Nei endonuclease VIII/NEIL1 and NEIL2 have been shown to protect the genome from the mutagenic consequences of 8-oxo-G damage. Finally, A:8-oxo-G base pairs are a substrate for MutY/MUTYH, which is the protein in the focus of this review. Information on the contribution of all of the other factors to genetic stability can be found in these detailed reviews (Lu et al., 2006a; Tsuzuki et al., 2007).

**Figure 1:**
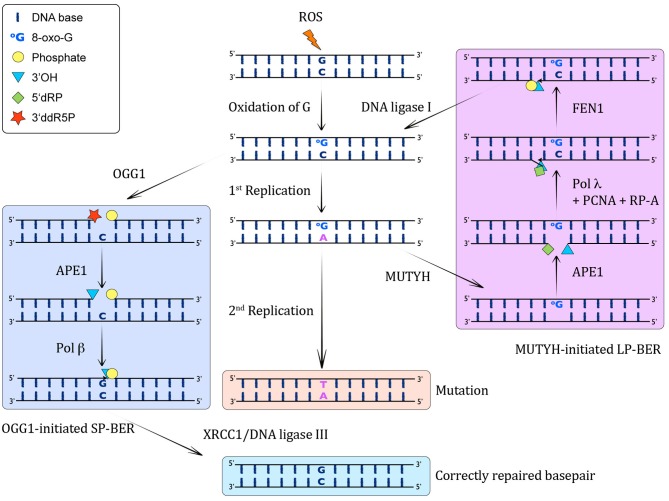
**MUTYH-initiated BER of A:8-oxo-G lesions.** When ROS attack DNA, they lead to the formation of C:8-oxo-G base pairs through oxidation of G. **Left column:** These can be recognized by OGG1, which excises the 8-oxo-G and incises the resulting AP-site by β-elimination, giving rise to a 3′ddR5P and a 5′P residue. This 3′ sugar phosphate is then removed by APE1, yielding in a 1 nucleotide gap with a 3′OH and a 5′P. Subsequently, pol β catalyzes the insertion of a G opposite the templating C in this SP-BER pathway, and ligation by XRCC1/DNA ligase I leads to restoration of an intact, correctly base-paired double-stranded DNA again. **Middle column:** If the C:8-oxo-G base pairs are not recognized before S-phase by OGG1, or they arise through oxidation in S-phase, the replicative pols will often incorporate a wrong A opposite 8-oxo-G, giving rise to A:8-oxo-G mispairs. If these are not corrected, another round of replication will lead to a CG→AT transversion mutation. **Right column:** The A:8-oxo-G base pairs can be recognized by MUTYH, which catalyzes the excision of the wrong A from opposite 8-oxo-G, leading to the formation of an AP site. This AP site is further processed by APE1, which results in a 1 nt gap with 3′OH and 5′dRP moieties. The incorporation of the correct C opposite 8-oxo-G and one more nucleotide is performed by pol λ in collaboration with the cofactors PCNA and RP-A, thus performing strand displacement of the downstream DNA strand. FEN1 cleaves the 5′ flap, leading to a 5′P moiety, which can be ligated by DNA ligase I to yield an intact C:8-oxo-G containing double-stranded DNA. This C:8-oxo-G is then again substrate for OGG1-mediated removal of 8-oxo-G **(left column)**.

In the *syn* conformation, 8-oxo-G functionally mimics the base pairing properties of a Thymine (T), which leads to the formation of stable A(*anti*):8-oxo-G(*syn*) Hoogsteen base pairs (David et al., 2007). Due to this particular behavior of 8-oxo-G, most pols often bypass 8-oxo-G lesions inaccurately by incorrectly inserting an A instead of the correct C, therefore giving rise to A:8-oxo-G mismatches (Maga et al., 2007). If these A:8-oxo-G mismatches are not repaired before the next round of replication, they can generate CG→AT transversion mutations that have the potential to transform cells and lead to cancer (Greenman et al., 2007). Oxidative damage to C:G base pairs in DNA leads to the generation of C:8-oxo-G base pairs. The majority of 8-oxo-G from these base pairs is recognized and removed from the genome by the OGG1 DNA glycosylase, which initiates a canonical short-patch base-excision repair (SP-BER) pathway involving apurinic endonuclease 1 (APE1), pol β, XRCC1, and DNA ligase III. This results in the restoration of the original C:G base pair [see Figure 1, Dianov et al., 1998; Fortini et al., 1999; Pascucci et al., 2002; Fromme et al., 2003 and reviewed in Van Loon et al. (2010)]. However, a problematic situation may arise when the replication fork encounters an 8-oxo-G. Such a scenario can result from either a failure of OGG1 to repair all 8-oxo-G lesions before the start of replication, or from oxidative stress during the S-phase. In contrast to UV-induced lesions, for instance, that present a block to the replicative pols (reviewed in Lehmann, 2002), 8-oxo-G is not considered a blocking lesion *per se* (Shibutani et al., 1991; Mozzherin et al., 1997; Avkin and Livneh, 2002). Nevertheless, it has been found that replicative pols (such as the Klenow fragment of *E. coli* pol I, calf thymus pol α and pol δ) show transient inhibition of chain extension 3′ to 8-oxo-G and extend promutagenic A:8-oxo-G base pairs more efficiently than the correct C:8-oxo-G base pairs (Shibutani et al., 1991; Einolf and Guengerich, 2001). Also, human pol δ has been demonstrated to stall at sites of 8-oxo-G lesions (Fazlieva et al., 2009). Very recently, we have proposed that a switch between the replicative pol δ and the repair pol λ promotes the correct bypass of 8-oxo-G lesions during replication (Markkanen et al., 2012a). Nevertheless, oxidative stress in context of DNA replication can result in the generation of A:8-oxo-G mispairs, which are substrates for MUTYH. As a monofunctional DNA glycosylase, MUTYH catalyzes the excision of the A mispaired with 8-oxo-G. Thus, MUTYH is a unique glycosylase as far as it removes an *undamaged* base from *opposite a DNA lesion*, instead of removing the damaged base. The steps following MUTYH-initiated repair of A:8-oxo-G lesions are discussed in more detail in the following. As this review is focused on MUTYH, the interested reader is referred to a detailed excellent review for more information on the cellular DNA glycosylases in general (Jacobs and Schar, 2012).

## Discovery

MutY, along with the other 8-oxo-G repair enzymes FpG and MutT, is phylogenetically an ancient protein, emphasizing the importance to cope correctly and efficiently with oxidative damage for living organisms (Jansson et al., 2010). MutY homologues have been identified in many organisms, both in prokaryotes as well as in eukaryotes. They all share the unique function of being able to remove an A that is incorrectly paired with 8-oxo-G, G, C, 5-hydroxyuracil (5-OH-U), or 2-hydroxyadenine (2-OH-A), as specified later on.

### Discovery of MutY in *E. coli*

The first mutators in *E. coli* strains were described about 60 years ago (Treffers et al., 1954) based on the observation that some strains showed an altered antibiotic resistance. These findings were used to engineer a systematic screening for mutators with certain properties. Nghiem et al. used Lac^−^ *E. coli* strains transformed with constructs encoding for β-galactosidase, each inactivated by a specific point mutation. When reverted back to Lac^+^ the specific base substitution reactivating the β-galactosidase could be identified. A strain with an increase in C:G→A:T transversion mutations revealed the so far not described locus called *mutY* to be responsible for the observed mutator phenotype (Nghiem et al., 1988).

In addition to the *mutY*, another locus, called *mutM*, was found to cause a change from C:G→A:T (Cabrera et al., 1988) when mutated and was later identified to encode the formamidinopyrimidine DNA glycosylase (Fpg) (Michaels et al., 1991). Neither *mutY* nor *mutM* strains showed a very pronounced phenotype on their own, but double mutant strains expressed an extremely high mutation rate (Michaels et al., 1992a). Mutations in *mutY* and *mutM* exclusively enhanced one type of transversion mutation, while neither frameshifts nor deletions were found, in contrast to what had been reported for other mutators (Nghiem et al., 1988).

It had been shown that the correction of A:G mispairs in *E. coli* extracts could occur by two distinct pathways: the methylation-dependent mutHLS mismatch-repair pathway that recognizes a variety of mismatches and repairs the unmethylated DNA strand, and a second methylation-independent mechanism specific to A:G mismatches (Su et al., 1988). Analysis of the second pathway revealed that the *mutY* gene product was involved in this novel DNA repair mechanism (Au et al., 1988). Cells defective in the mutHLS-dependent repair but proficient for *mutY* were still able to prevent C:G→A:T transversion mutations, and the *mutY*-dependent repair was dominant if both pathways were available. The function of the *mutY* gene product was finally elucidated by purification of a protein according to its ability to repair a A:G mismatch. The 36 kDa protein was capable of removing the mispaired base A from dsDNA and rendered the strand sensitive for cleavage by apurinic/apyrimidinic endonucleases at the site of the mismatch (Au et al., 1989). This result further underlined the hypothesis that mutY encoded for a DNA glycosylase, termed MutY, that initiated the repair of A:G mismatches while other mispairs, as for example A:C, were not recognized. Further on, Su et al. showed that MutY, with help of pol I and DNA ligase, was able to restore specifically A:G mismatches to C:G in a sequence independent manner (Su et al., 1988). Cloning and sequencing of the mutY gene finally revealed that it encoded for a 350 amino acids DNA glycosylase that could rescue the mutator phenotype of mutY *E. coli* strains (Michaels et al., 1990).

### Discovery of the mammalian MutY homolog (MUTYH)

The first experiments using cell extracts showed that, in general, humans had a repair mechanism for mismatches similar to those of bacteria preventing the generation of mutations during replication (Holmes et al., 1990; Thomas et al., 1991). The analysis of human HeLa nuclear extracts revealed the existence of two enzyme systems that could nick DNA specifically at sites of mispaired bases (Yeh et al., 1991). One of the identified systems showed a specific substrate recognition, cleaving the DNA at A:G mismatches and could be separated from other enzymes by chromatography. Since this enzyme showed the same substrate specificity as the bacterial MutY, Yeh et al. proposed to have identified its human homologue (Yeh et al., 1991).

The first characterization of a mammalian homologue of MutY was published by McGoldrick et al., who purified an enzyme from calf thymus that was acting on A:G mismatches. Apart from the substrate specificity they described several other features indicating that they had indeed purified a MutY homologue: An AP endonuclease activity was co-purified with the DNA glycosylase and the antibody generated against bacterial MutY recognized a band at the expected size and could inhibit the DNA glycosylase activity of the purified protein (McGoldrick et al., 1995). Based on the finding that CG→AT transversion mutations occur often in different kinds of cancer (Hollstein et al., 1991), the authors already hypothesized that the human MutY homologue might be involved in cancer prevention.

A few years after the characterization of human homologue of the 8-oxo-dGTP hydrolase MutT which removes 8-oxo-dGTP from the nucleotide pool (Sakumi et al., 1993), Slupska et al. succeeded in cloning and sequencing of the human *mutY* gene, termed *MUTYH* (Slupska et al., 1996). By screening different cDNA libraries for amino-acid sequence homologies, they identified a gene that showed 41% identity with the *E. coli mutY*. The gene was 7.1 kb long, contained 15 introns and encoded for a protein of 535 amino acids in length, which was consistent with the size of the protein that had been detected in HeLa cells (McGoldrick et al., 1995). By using *in situ* hybridization they could map the gene on chromosome 1, between *p32.1* and *p34.3*. The current status of knowledge is that the human *MUTYH* gene codes for at least 10 different isoforms of MUTYH protein. There are three major transcripts, α, β, and γ that differ from each other in the 5′ end sequence and are generated through alternative splicing (Ohtsubo et al., 2000). The transcript α3 was found to be the originally identified MUTYH, but so far it is not entirely clear what the functions of the different isoforms are and to which cell compartment they are localized, as we will discuss below in more detail.

## Nomenclature of MUTYH

Currently, literature referring to the protein product of the mammalian *MUTYH* gene is rather confusing due to a diversity of different synonyms and writing styles that have been used over the last years. The most commonly used names are MUTYH, MutYH, MYH, and hMYH. Here, we propose to uniformly use MUTYH as name for this protein in mammals in order to simplify the literature overview, because of the following reasons. Firstly, *MUTYH* [*MutY h*omolog (*E. coli*)] is the officially approved name for the gene from which MUTYH derives (HUGO Gene Nomenclature Committee). Secondly, the official protein name listed by leading protein databases (UniProtKB, neXtProt, Ensembl, and Reactome) is MUTYH. Thirdly, as the protein derives its name from the bacterial homolog mutY that was discovered first, the logical extension would be the addition of an “H” for “homolog” at the end of the protein name, which also leads to easy recognition of homology between MUTYH and MutY.

## Function of MutY and MUTYH

### MutY

#### MutY—substrate specificity

The currently known substrates for MutY and MUTYH are summarized in Table 1. Analysis of the substrate specificity for MutY demonstrated that it acts as a glycosylase on A:G, A:8-oxo-G, A:C, and A:8-oxo-A mismatches, always removing the undamaged A from each substrate (Michaels et al., 1992b). Lu et al. further refined the DNA determinants and substrate specificities for the catalytic activity of MutY, using binding and endonuclease assays with a variety of different A-containing mismatches, and concluded that DNA sequences proximal to the mismatch as well as specific functional groups of mismatched bases dictate the recognition and catalysis by MutY (Lu et al., 1995). Moreover, while MutY bound the A:8-oxo-G much tighter than A:G, its activity on A:8-oxo-G was weaker than on A:G mismatches. Bulychev et al. contradicted this notion in a subsequent report stating that A:8-oxo-G appeared to be the natural substrate for MutY, as judged by the specificity constants and the fact that the presence of an 8-oxo-group in G increased significantly the rate of removal of A from all tested substrates (Bulychev et al., 1996). Additionally to A:8-oxo-G, MutY was shown to bind to G:8-oxo-G mismatches as well, and it was capable of removing G from this substrate (Zhang et al., 1998). The sequence context surrounding an A:G mismatch was shown to also significantly influence the catalytic activity of MutY (Sanchez et al., 2003).

**Table 1:** **Substrate specificities of the different MutY and MUTYH proteins**.

**Protein**	**Base pair substrate**	**Excised base**	**References**
MutY *E. coli*	A:G	A	Michaels et al., 1992b; Lu et al., 1995; Gogos et al., 1996; Noll et al., 1999; Gu and Lu, 2001
	A:8-oxo-G	A	Michaels et al., 1992b; Lu et al., 1995; Gogos et al., 1996; Noll et al., 1999; Gu and Lu, 2001
	A:C	A	Michaels et al., 1992b
	A:8-oxo-A	A	Michaels et al., 1992b
	2-OH-A:G	2-OH-A	Hashiguchi et al., 2002; Pope and David, 2005
	2-OH-A:8-oxo-G	2-OH-A	Pope and David, 2005
	A:FapyG	A	Wiederholt et al., 2003
	G:8-oxo-G	G	Zhang et al., 1998
MutY *Th. thermophilus*	A:8-oxo-G	A	Back et al., 2006
	A:G	A	Back et al., 2006
	G:8-oxo-G	G	Back et al., 2006
	T:8-oxo-G	T	Back et al., 2006
MUTYH *S. pombe*	G:8-oxo-G	G	Doi et al., 2005
	A:8-oxo-G	A	Doi et al., 2005
MUTYH mouse	A:8-oxo-G	A	Tominaga et al., 2004; Pope and David, 2005
	A:G	A	Pope and David, 2005
	2-OH-A:G	2-OH-A	Pope and David, 2005
	2-OH-A:8-oxo-G	2-OH-A	Pope and David, 2005
MUTYH calf	A:G	A	McGoldrick et al., 1995; Parker et al., 2000
	A:8-oxo-G	A	McGoldrick et al., 1995; Parker et al., 2000
	A:C	A	McGoldrick et al., 1995; Parker et al., 2000
	G:8-oxo-G	G	Parker et al., 2000
	T:8-oxo-G	T	Parker et al., 2000
	C:8-oxo-G	C	Parker et al., 2000
MUTYH human	A:8-oxo-G	A	Slupska et al., 1999; Shinmura et al., 2000; Gu and Lu, 2001
	A:G	A	Slupska et al., 1999; Shinmura et al., 2000; Gu and Lu, 2001
	2-OH-A:G	2-OH-A	Ushijima et al., 2005

8-oxo-G is chemically labile toward further oxidation into guanidinohydantoin (Sp1), spiroiminodihydantoin (Sp2), oxaluric acid, and urea. Delaney et al. investigated the activity of MutY on these lesions by introducing them into single-stranded viral genomes which were replicated in *E. coli* proficient or deficient for MutY (Delaney et al., 2007). These lesions were found to be equally mutagenic in terms of frequency in both genetic backgrounds and to yield similar mutation spectra, suggesting that MutY does not play a role in the excision of these bases. Interestingly Sp1 and Sp2 were more toxic to the cells that were proficient in MutY.

2-hydroxyadenine (2-OH-A) is a lesion that is induced by Fenton-type ROS and is produced for instance by H_2_O_2_ treatment of cultured mammalian cells (Jaruga and Dizdaroglu, 1996). Incorporation of 2-OH-dATP into the bacterial genome by pol III was shown to yield slightly increased mutant frequencies in a MutY deficient background in *E. coli*, suggesting that the processing of 2-OH-A damage possibly also involves the action of MutY (Kamiya and Kasai, 2000a). However, follow-up work by the same authors showed that, irrespectively of the base in the complementary strand, DNA with 2-OH-A presented a very poor substrate for MutY, and therefore illustrated that neither MutY nor Fpg seemed to play a role in 2-OH-A removal from DNA (Kamiya and Kasai, 2000b). Another result by Hashiguchi et al. again reassessed this finding and they reported that MutY indeed bound to 2-OH-A in duplex with G, A, or C and displayed a DNA glycosylase activity capable of removing 2-OH-A from 2-OH-A:G mismatches, which was dependent on the C-terminal domain of the protein (Hashiguchi et al., 2002).

FapyG is a DNA lesion that arises from oxidative stress by ring-fragmentation of the purine base. MutY excised A from A:FapyG mismatches, and this reaction was faster than the removal of A from A:G, but still slower than that from A:8-oxo-G *in vitro* (Wiederholt et al., 2003).

One group reported that MutY efficiently recognized 7-deaza-2′-deoxyadenosine (Z) and its non-polar isostere 4-methylindole-beta-deoxynucleoside (M) opposite 8-oxo-G and G in DNA, with a preference for M:8-oxo-G over Z:8-oxo-G mispairs (Chepanoske et al., 2000b). This finding was contradicting a previous report, in which Z:G mispairs were neither bound nor processed by MutY (Lu et al., 1995).

Lu et al. showed that MutY competes with and inhibits endonuclease VIII on its natural substrate, the hydroxyurea (hoU):A mismatch (Lu et al., 2006b).

A MutY variant from *Thermus thermophilus* processed A:8-oxo-G, G:8-oxo-G as well as T:8-oxo-G and A:G mismatches, but in contrast to other MutY variants, was shown to harbor a bifunctional glycosylase activity (Back et al., 2006).

#### MutY—enzymatic activity

The cloning of *E. coli* MutY revealed that it shared significant sequence homology to the bacterial endonuclease III (EndoIII), which acts on damaged base pairs (Michaels et al., 1990). MutY was shown to be an iron-sulfur (Fe-S) cluster protein containing both N-glycosylase and a 3′ AP endonuclease activity (Tsai-Wu et al., 1992). Initially there was some confusion regarding the enzymatic activity of MutY. While some reports stated that MutY also acted as an endonuclease on AP sites, therefore functioning as a bifunctional glycosylase (Tsai-Wu et al., 1992; Lu et al., 1995, 1996; Gogos et al., 1996; Manuel and Lloyd, 1997), Zharkov and Grollman showed that MutY does not harbor any AP lyase activity (Zharkov and Grollman, 1998). They hypothesized that the previous observations for the observed AP-activity were rather caused by heat-induced cleavage of the AP site and not due to an actual enzymatic activity. Moreover, this report suggested that the tight binding of MutY to its DNA substrate prevented the access of another bacterial glycosylase, the formamidopyrimidine-DNA glycosylase (Fpg), to the substrate. Consequently, MutY seemed to prevent a possible generation of a DNA double-strand break (DSB) by Fpg and thus possibly to play a role in the regulation of BER.

#### MutY—catalytic mechanism

When considering the catalytic activity of MutY (or any other DNA glycosylase), it is important to keep in mind that the catalytic cycle can be roughly subdivided into different stages, namely (1) recognition and binding of the enzyme to the substrate, (2) hydrolysis of the N-glycosidic bond or base-excision, and (3) dissociation of the enzyme or release of the resulting AP site. We have tried to structure the discussion according to these three steps in the catalytic cycle, whenever possible.

***Substrate recognition.*** Multiple studies elucidating the contributions of the different parts of the MutY protein have been undertaken. Proteolytic digestion of MutY with thermolysin produced two fragments, an N-terminal one of 25 kDa and a C-terminal one of 12 kDa, respectively (Gogos et al., 1996). While the 12 kDa fragment did not display any detectable enzymatic activity, it was found to play an important role in the repair of mismatched oxidized DNA, as its deletion significantly impaired the binding and activity of MutY on A:8-oxo-G substrates, while it did not influence binding and cleavage of A:G substrates. On the other hand, a similar study, generating a 26 kDa N-terminal domain of MutY by trypsin-mediated proteolysis showed that this 26 kDa subunit was catalytically active, contained both DNA glycosylase and AP lyase activity, and was functionally identical with the full-length protein (Manuel et al., 1996; Manuel and Lloyd, 1997). A 14 kDa C-terminal domain of MutY (AA 1–226) was demonstrated to be the principal determinant for 8-oxo-G specificity, as its deletion remarkably enhanced the dissociation of the enzyme from A:8-oxo-G and reduced the rate of A removal from these substrates compared to A:G mismatches (Noll et al., 1999). This was interpreted such that the C-terminal domain facilitated A base flipping. Also, this study found that the C-terminal domain of MutY showed homology with MutT, suggesting that it might serve in 8-oxo-G recognition. Another report supported this view by showing that the N-terminal domain of MutY (AA 1–226) had a 18-fold lower affinity for binding various 8-oxo-G mismatches, a reduced catalytic preference for A:8-oxo-G over A:G mismatches and exhibited a lower inhibition on Fpg activity than the wild-type (wt) MutY (Li et al., 2000). These results suggested that the C-terminal domain of the protein determines its 8-oxo-G specificity and is crucial for mutation avoidance. The C-terminal domain was then shown to mediate additional contacts between MutY and A:8-oxo-G containing substrates that are not found in interaction with A:G (Li and Lu, 2000), thereby promoting the efficient recognition of substrates by MutY (Chmiel et al., 2001) and also affecting the catalytic activities toward A:G mismatches (Li and Lu, 2003). Taken together, the C-terminal domain of MutY seems to contribute substantially to the A:8-oxo-G substrate recognition.

It is still not entirely clear, how MutY is capable to efficiently recognize all its substrates from among the vast amount of undamaged base pairs. Along this line, the Fe-S cluster present in MutY was shown to be critical for the specific recognition of its DNA substrate and its enzymatic activity (Porello et al., 1998a; Golinelli et al., 1999; Chepanoske et al., 2000a). It has also been suggested that the relative oxidation resistance of the Fe-S cluster may be an important aspect to guarantee the activity of MutY under conditions of oxidative stress (Messick et al., 2002). K142 in MutY, earlier shown to be involved in formation of tight interactions with DNA, was shown to make specific contacts with 8-oxo-G, and DNA-mediated charge transport (CT) was suggested as signal to promote the binding of MutY to DNA from a distance (Boon et al., 2002). Along this line, DNA-mediated CT led to oxidation of DNA-bound MutY, suggesting that G radicals provide the signal to stimulate DNA repair by the redox activation of DNA repair proteins through CT (Yavin et al., 2005). Further substantiating this idea, Boal et al. proposed that the rapid redistribution of proteins to the sites of DNA damage was mediated through redox activation involving the Fe-S clusters in proteins such as MutY and EndoIII (Boal et al., 2005; Yavin et al., 2006). A theoretical study of the DNA damage recognition by *Bacillus stearothermophilus* MutY proposed that the CT from MutY to DNA through hole transfer, which is specially efficient near an 8-oxo-G, leads to the stabilization of the enzyme in a conformation required for recognition of the lesion (Lin et al., 2008). Examination of the charge-transfer model by atomic force microscopy further validated this concept and emphasized the possibility that indeed repair proteins might be recruited to DNA lesions by DNA-mediated CT in the cellular context (Boal et al., 2009). The authors therefore proposed a model wherein the binding of Fe-S cluster containing DNA repair proteins (such as MutY and EndoIII) to DNA activates them toward oxidation. First, the formation of a guanine radical oxidizes a repair protein bound to DNA and thus stabilizes the binding of this protein. This step is followed by the binding of a second protein near the first one. Because also this protein gets oxidized during binding and transfers an electron to the DNA, it will induce a DNA-mediated CT from the second to the first protein if no damage is present in the DNA stretch between the two binding sites. This CT leads to reduction of the first protein and thus to its release from DNA, because in the reduced state it has a lower affinity to DNA. However, if there is a DNA lesion between the two bound proteins, the CT does not take place (it is “blocked” by the intervening lesion). In this situation both of the proteins remain bound and can subsequently catalyze repair steps. Through examination of CT mutants of EndoIII the group subsequently linked the ability of a repair protein to carry out DNA CT and its ability to localize to damaged DNA and thus further underlined their model (Romano et al., 2011). Taken together, the role for the Fe-S cluster as redox cofactor to search for damaged bases using DNA-mediated CT becomes more and more substantiated and really presents a plausible scenario to explain the mechanisms of full-genome search for lesions.

***Base-excision.*** Investigations into the glycosylase activity of MutY revealed a distinctive difference in the processing of A:8-oxo-G compared to A:G mismatches (Porello et al., 1998b). Hydrolysis of A from opposite 8-oxo-G was at least 6-fold faster than from the A:G mispair. Interestingly however, MutY “lingered” when excising from an A:8-oxo-G base pair and released the product with a much slower kinetic compared to the A:G substrate. This delay in substrate release might protect 8-oxo-G from being prematurely accessed and removed by other glycosylases, as also suggested by Zharkov and Grollman (1998). A detailed study of the active site revealed the importance of several amino acids involved in the glycosylase as well as DNA binding activities of MutY (Wright et al., 1999). Bifunctional glycosylases all bear a conserved lysine residue believed to be important for the initial nucleophilic attack in base removal near their active site, which is lacking in their monofunctional counterparts. To yield more insight into the role of this residue on a structural basis, Williams et al. investigated whether insertion of such a lysine residue into the catalytic site of MutY had any influence on its activity. Indeed, a point-mutation at S120K generated a MutY mutant capable of catalyzing DNA strand scission at a rate that was similar to its A excision activity from A:G and A:8-oxo-G substrates, and also changed it into a bifunctional glycosylase (Williams and David, 2000). This study illustrated that the basic mechanisms of mono- and bifunctional glycosylases were quite similar. The glycosylase activity of MutY was shown to involve a Schiff base intermediate, characteristic for other bifunctional DNA glycosylases that catalyze a β-lyase reaction, though no β-lyase step (*per se* only performed by bifunctional glycosylases) could be observed (Williams and David, 1998). Reduction of this Schiff-base intermediate with borohydride resulted in the formation of a covalent MutY-DNA adduct. To identify the residues involved in this covalent complex formation, Williams et al. constructed different MutY mutants and identified K142 to be the primary residue for such covalent associations (Williams and David, 1999). As the DNA binding and enzymatic activity of the K142A mutant was comparable to that of the wt enzyme, the formation of this covalent intermediate was not required for removal of A and was suggested to be a consequence of the unusually high affinity of MutY for the product of its glycosylase activity. Similarly, mutation of K142 to glutamine in MutY was shown to also abrogate its ability to form a Schiff base with DNA, while still retaining some of its catalytic activity (Zharkov et al., 2000). Interestingly, this mutation selectively impaired the processing of A:G base pairs, but not of A:8-oxo-G substrates, primarily by interfering with the binding to A:G substrates, but did not impair the catalytic activity *per se*, again confirming that it was not directly involved in the catalytic step. Using unnatural substrates to elucidate the tolerance of MutY to different modifications of the A or the 8-oxo-G in mismatches in an *E. coli*-based cellular assay, it was seen that, while modification of A was tolerated rather well, modification of 8-oxo-G resulted in a drastic reduction of base-excision (Livingston et al., 2008). This led to the conclusion that the presence of 8-oxo-G is critical for MutY to recognize A:8-oxo-G mismatches *in vivo* to initiate repair. D138 and Q37 are both residues that are involved in the catalytic mechanism of MutY-mediated A removal. Interestingly, their substitution yielded mutants with a range of different excision activities. Studies of these mutants demonstrated that changes which reduced the excision activity were better tolerated and less compromising to A:8-oxo-G repair *in vivo* in *E. coli* than those affecting the recognition of A:8-oxo-G mismatch affinity (Brinkmeyer et al., 2012). Therefore, this report suggested that the recognition of A:8-oxo-G mismatches was more important for the correct repair of these duplexes than the actual glycosylase activity *per se*. Interestingly, this can be reconciled with the fact that the release of the substrate by MutY after base-excision is much slower than the actual N-glycosidic activity, seemingly demonstrating that the rate-limiting step of this enzyme is rather the identification of its substrate than the excision step itself. Additionally, this study also revealed which residues are critical for the selectivity and specificity of MutY.

***Substrate release.*** The product release rate of MutY could be greatly enhanced by the two proteins AP-endonuclease IV and exonuclease III, and this effect depended on the presence of the C-terminal domain of MutY (Pope et al., 2002). Also, endonuclease VIII was found to promote MutY dissociation from AP:G substrates, but not from AP:8-oxo-G, and to further process these by βδ elimination (Lu et al., 2006b). This study also showed that MutY interacts with endo VIII through its C-terminus and competes with endo VIII on its natural substrate, the hydroxyurea (hoU):A mismatch, thus inhibiting its activity and possibly reducing the mutagenic effects of hoU. Taken together, it seems important that also the substrate release step is tightly regulated, in order to orchestrate the following steps and to protect the 1-nt gap resulting from base-excision.

#### Structure of MutY and the removal of adenine opposite 8-oxo-G

**Figure 2:**
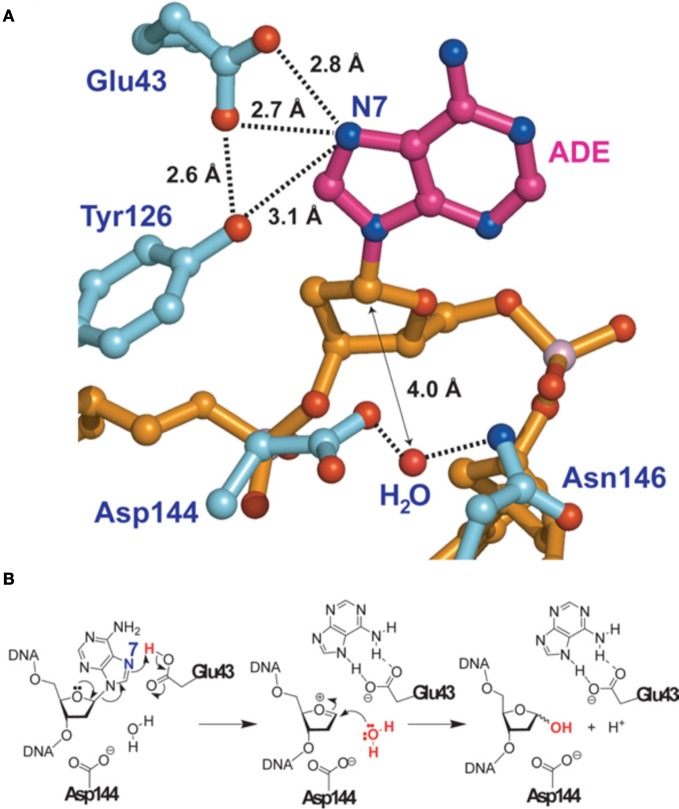
**Adenine removal by MUTYH. (A)** View of the substrate adenosine interacting with catalytic residues of MUTYH. **(B)** Proposed glycolytic mechanism based on the structural information of **(A)**. Reproduced form Lee and Verdine (2009). For details see text.

The most precise structure of MutY comes from studies with *Bacillus stearothemophilus* (Lee and Verdine, 2009) (Figure 2). After binding to the 8-oxo-G:A mispair MutY flips out the A from the DNA double-helix. A water molecule is positioned between Asp144 and Asn146 in the MutY lesion-recognition pocket of the enzyme. Earlier studies included biochemical and computational studies on uracil DNA glycosylase (Werner and Stivers, 2000; Dinner et al., 2001) suggested that a so called dissociative action occurs, where the cleavage of the N-glycosylic bond and the subsequent attack of the water molecule on the C1′ (arrow in Figure 2A) do not occur simultaneously, but rather in two discrete steps. In addition Glu43 can adopt a so-called bifurcated hydrogen-bonding interaction of 2.7 and 2.8 Angströms, respectively, with N7 of A (Figures 2A,B). These short distances together with a protonated Glu43, provides acidity and therefore full hydrogen bonding to the N7 of A. As indicated in Figure 2B such a conformation favors the scission of the glycosylic bond between A and the deoxyribose. A similar structure has also been identified for human MUTYH, for which a fragment lacking the first 64 amino-acids was crystalized (Luncsford et al., 2010).

The structure of MutY catalytic core revealed that the two helical domains form a positively-charged groove, positioning the A-binding pocket at their interface (Guan et al., 1998). Also, this study confirmed a nucleotide flipping mechanism by a substitution of the Watson–Crick hydrogen bond partners by protein atoms. Recognition of 8-oxo-G seems to occur independently of double-stranded DNA or of an A-mismatch, and sequential extrusion of 8-oxo-G followed by A occurs in MutY, as demonstrated by Bernards et al. (2002). MutY has been proposed to assemble into a dimer upon substrate binding to yield an active form of the enzyme (Wong et al., 2003). This idea was further substantiated by a study that suggested a model for MutY binding of the mismatched DNA that involves scanning of the DNA by one molecule which enhances binding of second MutY molecule upon encountering an A:8-oxo-G mismatch (Lee et al., 2004).

Kinetically, it has been suggested that the release of A happens fast, while the rate-limiting step was the release of the AP-site (McCann and Berti, 2003). Further investigations into the transition state structure of MutY showed that the irreversible breakage of the N-glycosidic bond could not take place until a H_2_O atom was present and that the enzyme stabilized the excision site after excision (McCann and Berti, 2008). Recently, a two-step reaction was proposed to be the basis of the catalytic activity of MutY, as opposed to the three-step mechanism proposed before (Tiwari et al., 2011).

Investigations of the roles of the different H_2_O molecules involved in catalysis by MutY from *B. stearothermophilus* and *E. coli* suggested that E43 and N7 may be important factors for the activity of MutY (Brunk et al., 2012). Further insight into the roles of the substrate A residues N7 and N3 during catalytic excision by MutY have been gained recently (Michelson et al., 2012).

#### MutY in living cells

In *E. coli*, MutY was shown to be co-transcribed as first gene of a part of a large operon, together with Fpg, the bacterial DNA glycosylase which removes 8-oxo-G from the DNA (Gifford and Wallace, 1999). This further emphasized the involvement of MutY in the repair of 8-oxo-G base pairs in an interplay with Fpg and thus in the response to oxidative DNA damage. Somewhat surprisingly at first glance, oxidative stress down-regulated the activity of MutY by 70% as well as its mRNA levels, and in contrary it was induced more than 2-fold under anaerobic conditions (Yoon et al., 2003). This negative regulation of MutY was mediated by the regulatory genes fur, fnr and arcA. These results were explained with the idea that MutY activity had to be restrained when increased incorporation of 8-oxo-dGTP could possibly take place, which is during times of oxidative stress. This is important, because 8-oxo-dGTP could be inserted opposite a correct templating A, which might erroneously get excised by the action of MutY, thus actually acting *promutagenic* in this scenario instead of protecting from mutations taking place.

Screening for mutator loci leading to GC→CG transversions in *E. coli*, Zhang et al. found that inactivation of MutY led to accumulation of these mutations (Zhang et al., 1998). As mentioned above, they showed that MutY bound to G:8-oxo-G mismatches and was capable of removing G from the G:8-oxo-G mispair.

To analyze the impact of *mutT, mutM* (which encodes the Fpg DNA glycosylase that removes 8-oxo-G from C:8-oxo-G base pairs in bacteria), and *mutY* on the mutational spectra, following considerations have to be taken into account. In the context of 8-oxo-G and 8-oxo-dGTP (1) CG→AT mutations can arise either from oxidation of C:G to C:8-oxo-G or from incorporation of 8-oxo-dGTP opposite C, followed by wrong incorporation of A opposite 8-oxo-G by the replicative pols during the next round of replication. (2) AT→CG mutations are based on incorporation of 8-oxo-dGTP opposite templating A. Analyzing different combinations of mutated strains in *mutT, mutY*, and *mutM*, Fowler et al. found that (1) *mutT* does not increase CG→AT transversions, regardless of the *mutY* and *mutM* background, suggesting that 8-oxo-dGTP does not often get incorporated opposite C but rather opposite A. (2) AT→CG transversions are reduced in *mutY* and *mutMmutY* backgrounds, suggesting templating 8-oxo-G preferentially pairs with dATP, which then is a substrate for MutY to excise A from the A:8-oxo-G pair, followed by Fpg that removes 8-oxo-G paired with C. And finally (3) *mutY* and *mutMmutY* decrease AT→CG mutations (arising from incorporation of 8-oxo-dGTP opposite templating A) in a *mutT wt* background, suggesting that a certain amount of 8-oxo-G gets incorporated into DNA even in the presence of functional MutT (Fowler et al., 2003). No strand bias in the mutation rate between leading and lagging strand synthesis in either a *mutMmutY* or a *mutT* background could be detected in *E. coli* using a supF shuttle vector (Watanabe et al., 2001). Interestingly, Bridges et al. showed that the rate of mutation markedly increased in starved *mutY* mutant *E. coli*, yielding CG→AT transversion mutations (Bridges et al., 1996). This phenotype could be further enhanced by additional mutation of *mutM*, even though mutation of *mutM* alone did not cause this effect. Also, addition of catalase to the plates did not alter the mutation rates, indicating that extracellular H_2_O_2_ was not involved in the generation of mutations, and it was suggested that singlet oxygen could be the source of internal DNA damage. These findings indicated that MutY may regulate the activity of Fpg in resting cells. Expression of MutY from a *mutY-lacZ* fusion construct was shown to be enhanced under aerobic compared to anaerobic conditions, but not to be down-regulated by nutrient limitation (Notley-McRobb et al., 2002). However, in many cases, nutrient limitation led to *mutY* inactivation by deletion, suggesting it might serve as a mechanism to increase mutation rates under these adverse conditions.

Clustered lesions, as induced by ionizing radiation, are defined as two or more lesions formed within one to two helical turns of the DNA. They present a challenge to the repair machinery of the cell. An 8-oxo-G in the vicinity of an AP site was found to retard the processing of the AP site by endo III and Fpg, and the AP site was found to elevate the mutation frequency at 8-oxo-G in *wt, nth, fpg*, and *mutY* deleted *E. coli* (Cunniffe et al., 2007). Interestingly though, the mutation frequency in *mutYfpg* null cells was reduced by the presence of the AP site, suggesting that the processing of tandem lesions challenges the cellular repair machineries. Similar findings by Noguchi et al. investigating the interplay of 1-nt-gaps and 8-oxo-G lesions in clusters in *E. coli* demonstrated again, that the mutagenic potential of 8-oxo-G depends on the presence and the position of the gap (Noguchi et al., 2012).

MutY competed with MutS-dependent mismatch-repair when A:C mispairs were present, especially in the presence of an increased dCTP pool (Kim et al., 2003). In *E. coli*, MutY has been shown to interact via its Fe-S cluster with the ATPase domain of MutS, which enhanced the binding affinity of MutY to A:8-oxo-G mismatches (Bai and Lu, 2007). MutY expression and activity were enhanced in a *mutS* mutant strain, and AT→GC transversions were reduced by additional mutation of mutY in a mutS background, suggesting a cooperative effect of MutY and MutS in repair of 8-oxo-G damage. Analysis of *Bacillus subtilis* revealed that the expression of MutY increased drastically upon deletion of *mutSL* operon in starved cells, possibly to disturb the balance between MutY and MMR proteins to support the production of mutations, which might give growth advantages to these cells (Debora et al., 2011).

In *Streptococcus mutans*, an oral pathogen, strains with mutations of *mutY* were shown to display elevated mutation rates, increased resistance to killing by acid and oxidative agents as well as higher virulence compared to the parent strain, suggesting that loss of a BER factor such as MutY could confer an advantage to pathogenic organisms (Gonzalez et al., 2012).

#### MutY and BER in *E. coli*

Reconstitution experiments with purified proteins from *E. coli* revealed, that the presence of Ape1, pol I, and DNA ligase is sufficient to catalyze the entire repair pathway of G:A mismatches *in vitro* (Au et al., 1989). Further elucidation of the pathway was achieved, when Radicella et al. showed that the average repair tract length initiated by MutY in *E. coli* is 9–27 nucleotides long, starting at the removed A, and involved pol I, even though the involvement of other pols was also evident (Radicella et al., 1993). This finding was further refined *in vitro*, when Tsai-Wu et al. found pol I to be responsible for generating these tracts of 5–12 nucleotides length (Tsai-Wu and Lu, 1994).

### MUTYH

#### MUTYH activity and substrate specificity

The substrate specificities for MUTYH are summarized in Table 1. The mammalian homolog of MutY, MUTYH, was first purified from calf thymus and catalyzed removal of A from A:G, A:8-oxo-G and A:C mismatches (McGoldrick et al., 1995). Subsequently, expression and purification of the cloned human protein confirmed its activity to remove A from A:8-oxo-G and A:G base pairs *in vitro*, supporting that also the human homolog is a *bona fide* DNA glycosylase (Slupska et al., 1999). Purification of MUTYH from calf liver mitochondria yielded a protein that complexes with A:8-oxo-G, G:8-oxo-G, and T:8-oxo-G, weakly with C:8-oxo-G but not with A:G and A:C mismatches and removed A mispaired with G, C, or 8-oxo-G while weakly removing G from G:8-oxo-G mispairs (Parker et al., 2000). Purification of the murine MUTYH revealed strong similarities to MutY function, even though the intrinsic rates of A removal were lower than by MutY (Pope and David, 2005). Shinmura and colleagues reported that both the purified nuclear and mitochondrial recombinant isoforms of human MUTYH were active, and predominantly removed A from A:8-oxo-G mispairs rather than A:G mispairs under physiological salt concentrations (Shinmura et al., 2000). MUTYH in human cell extracts was shown to be more active in binding and glycosylase activity toward A:G mismatches than recombinant MUTYH expressed in bacteria (Gu and Lu, 2001). Furthermore, the authors found this native form of MUTYH to migrate slower on a non-denaturing polyacrylamide gel than recombinant human MUTYH purified from bacteria. Moreover the native form seems to be phosphorylated, thus apparently enhancing its glycosylase activity predominantly on A:G but also on A:8-oxo-G. As the phosphorylation status of MUTYH did not alter its electric mobility, it was suggested to be possibly associated with other proteins to account for the higher apparent molecular weight. Accordingly, co-migration of APE1 and MUTYH with A:8-oxo-G substrates could be identified. Ohtsubo et al. found that MUTYH likely also harbors an activity to remove 2-OH-A (Ohtsubo et al., 2000). Removal of 2-OH-A from opposite 8-oxo-G or G has been described for murine MUTYH (Pope and David, 2005) and was confirmed for human MUTYH as well (Ushijima et al., 2005). MUTYH from *S. pombe* was able to remove G from G:8-oxo-G mismatches as efficiently as A from A:8-oxo-G mismatches, and its expression reduced the frequency of GC→CG transversions in an *E. coli mutY* mutant, suggesting it might be involved in the repair of G:8-oxo-G lesions (Doi et al., 2005).

A:8-oxo-G substrates processed by murine MUTYH were protected from inappropriate access by OGG1 and APE1, thus preventing the formation of DSBs (Tominaga et al., 2004).

A study by Miyako et al. found that mitochondrial DNA (mtDNA) from HeLa cells could be cleaved by recombinant *E. coli* MutY, in contrast to Fpg which has been shown to barely cleave mtDNA (Driggers et al., 1993; Hegler et al., 1993), and that this cleavage took place roughly at a rate that was expected to correspond to the amount of 8-oxo-G present in endogenous mtDNA (Miyako et al., 2000). Suzuki et al investigated the repair of 8-oxo-G in DNA and 8-oxo-dGTP in 293T cells using supF shuttle plasmids (Suzuki et al., 2010). While knockdown of OGG1, MUTYH, NTH1, and NEIL1 all led to a significant increase in CG→AT transversions caused by the C:8-oxo-G pair in the shuttle plasmid, only knockdown of MUTYH resulted in a reduction in AT→CG transversions induced by 8-oxo-dGTP. In summary, MUTYH displays remarkable similarity to its bacterial homolog MutY regarding its activity and substrate specificity.

#### Localization of MUTYH

The subcellular localization of MUTYH was rather enigmatic for a long time. A study using expression of tagged proteins in COS-7 cells revealed that MUTYH was mainly transported to the mitochondria, which was probably the result of the isoform that was used (Takao et al., 1998). Follow-up work by the same group identified an alternatively spliced transcript differing in exon 1, leading to the nuclear localization of this variant (Takao et al., 1999). Ten further isoforms containing unique 5′sequences that could be grouped into three types were subsequently described, and suggested to encode multiple authentic MUTYH proteins (Ohtsubo et al., 2000). Other reports have further discussed the localization of MUTYH in cells, finding isoforms targeted to the nucleus (Tsai-Wu et al., 2000; Ichinoe et al., 2004) or the mitochondria (Englander et al., 2002; Ichinoe et al., 2004). However, work still needs to be done to analyze the distribution of isoforms to the different subcellular compartments in different cell and tissue types to clarify this matter further.

Analyzing the distribution of endogenous MUTYH in serum-stimulated proliferating MRC5 cells with antibodies, Boldogh et al. reported both nuclear and mitochondrial localization of MUTYH (Boldogh et al., 2001). The nuclear form co-localized with BrdU and the proliferating cell nuclear antigen (PCNA) and, similarly to PCNA, increased 3- to 4-fold to peak during S-phase compared to G1, whereas levels of OGG1 or MTH1 did not change during the cell cycle. These studies suggested a role of targeting MUTYH to the replication fork to ensure that its activity is directed to the newly synthesized template strand. Subsequently, DNA replication was shown to enhance the MUTYH-dependent repair of A:8-oxo-G mismatches *in vivo*, and it was demonstrated that the interaction with PCNA was critical for the activity of MUTYH (Hayashi et al., 2002). Taken together, these findings clearly support a replication-associated function of MUTYH.

#### MUTYH and DNA damage signaling

Recently, a number of reports have accumulated that link MUTYH to the DNA damage response and implicate it in apoptotic signaling. To investigate the contribution of nuclear and mitochondrial accumulation of oxidative base lesions to the triggering of apoptosis, Oka and colleagues used *OGG1* knockout (ko) cells deficient in the nuclear or mitochondrial form of MUTYH, respectively (Oka et al., 2008). The accumulation of single-strand breaks in nuclear DNA triggered PARP-dependent cell death and could be rescued by depletion of nuclear MUTYH. The same was true for mitochondria, where MUTYH triggered calpain-dependent cell death by single-strand breaks. These results suggested that MUTYH catalyzes the formation of single-strand breaks in both of these DNAs, hence leading to the execution of apoptosis. Exposure of human cells to sodium nitroprusside, an agent that causes 8-oxo-G accumulation in cellular DNA by acting as an NO donor, led to MUTYH-dependent cell death that was initiated by oxidized bases in the mitochondrial, but not the nuclear DNA (Ichikawa et al., 2008). The role of single-strand breaks generated by MUTYH in the induction of cell death was further underlined by the finding that synthetic sickness/lethality mediated by either inhibition of pol β combined with MSH2, a component of the mismatch repair pathway, or pol γ with MLH1, both of which led to a nuclear 8-oxo-G accumulation, could be rescued by silencing of MUTYH (Martin et al., 2010). BER has been implicated in many different pathological conditions of the central nervous system (reviewed in Bosshard et al., 2012). A very recent report implicated MUTYH in degeneration by triggering apoptosis in microglia and neurons through initiation of single-strand breaks during repair of A:8-oxo-G mismatches (Sheng et al., 2012). Nuclear accumulation of 8-oxo-G triggered PARP-dependent apoptosis in microglia, while mitochondrial 8-oxo-G accumulation led to calpain-dependent apoptosis in neurons. All these findings are in agreement with a model, wherein the repair of DNA mismatches by MUTYH leads to generation of toxic single-strand breaks, and thus contributes to cellular death in case of excessive damage burden (i.e., an amount of DNA damage that surpasses the cellular capacity to further process these lesions properly). Thus, this model explains, why under conditions of severe damage the *absence* of MUTYH is *beneficial for the survival* of the cells. On the other hand, there are a number of reports that show that *loss* of MUTYH actually can *sensitize* cells to DNA damaging agents. Along this line, double mutations in *S. pombe MUTYH* with *RAD1* or, to a lesser extent *RAD9*, were shown to enhance the sensitivity of the cells to DNA damaging agents and hydroxyurea (Jansson et al., 2008). The consequences of these deficiencies were chromosome segregation defects and checkpoint failure after UV irradiation, as well as morphological defects, even in the absence of DNA damaging agents. This implicated MUTYH in the repair of a wide range of DNA damage and linked it to the checkpoint pathway. Under low-dose oxidative stress, *MUTYH OGG1* double-ko mouse cells also showed hypersensitivity to oxidation damage and a reduction of S phase concomitant with an increase of G2/M phase cells, while the levels of cell death remained unchanged (Xie et al., 2008). Furthermore, an increase in centrosome amplifications and formation of multinucleated cells could be observed in the surviving fraction of the ko cells, suggesting an involvement of MUTYH and OGG1 in the regulation of cell-cycle progression and cell division under oxidative stress. Further evidence implicating MUTYH in checkpoint control came from a study showing that siRNA-mediated knockdown of MUTYH resulted in a decreased phosphorylation of ATR and Chk1 upon treatment of HEK293 cells with HU or UV (Hahm et al., 2011). Concomitantly, the authors observed an increase in the phosphorylation of Cdk2 as well as the amount of the Cdc25A phosphatase, suggesting that MUTYH was involved in activation of the DNA damage response.

Thus, there seems to be growing evidence that implicates MUTYH to be an important factor in the cellular response to oxidative stress and inflammatory conditions by an involvement in cell death signaling (as discussed in Oka and Nakabeppu, 2011). Along these lines, MUTYH has been suggested to play a role in mitochondrial dysfunction in the pathogenesis of Parkinson's disease (Fukae et al., 2007; Nakabeppu et al., 2007). However, it still remains to be clarified how MUTYH can initiate apoptosis of cells in some instances, while it seems to protect from apoptosis in others.

#### Impact of MUTYH knockout on oxidative DNA damage and tumorigenesis *in vivo*

The data on cells and mice with biallelic deletion of MUTYH are somewhat discrepant. MUTYH ko embryonic stem cells displayed a mutator phenotype, but did not show any hypersensitivity toward oxidative stress induced by H_2_O_2_ or menadione (Hirano et al., 2003). A study with *mutyh*^−/−^ knockout mice by Xie et al revealed no significant increase in survival or tumor incidence after 14 months, suggesting that MUTYH deficiency is not sufficient to cause a tumor-predisposition (Xie et al., 2004). This study also showed that combined ko of MUTYH with OGG1 led to a decrease in life span and increased tumor formation for double ko mice compared to single ko. Interestingly, 75% of the lung tumors showed an activating GC→TA transversion mutation at codon 12 of K-ras, a feature that is often detected in MUTYH-associated polyposis (MAP) tumors, but none in the p53 gene or in the adjacent normal tissues. Additional heterozygosity for Msh2 (*mutyh*^−/−^ *ogg1*^−/−^ *msh2*^±^) did not inflict on the total tumor incidence but accelerated malignant lung and ovarian tumor formation in the *mutyh*^−/−^ *ogg1*^−/−^ background. A complete knockout of Msh2 to generate triple ko (*mutyh*^−/−^ *ogg1*^−/−^ *msh2*^−/−^) further increased tumor incidence and decreased survival time, but did not differ from the phenotype displayed by *msh2*^−/−^ single knockouts. This was suggested to be due to the strong mutator phenotype of *msh2*^−/−^ mice that might mask additional difference caused by *mutyh*^−/−^ and *ogg1*^−/−^.

Spontaneous mutagenesis in the small intestine of *ogg1*^−/−^ and *mutyh*^−/−^ *ogg1*^−/−^ double deficient mice at the age of 4–5 weeks using a transgene reporter revealed increased mutations in the double-ko's but not in the *ogg1*^−/−^ mice (Isogawa, 2004). Furthermore, the GC→TA mutation frequency increased in *mutyh*^−/−^ and in *ogg1*^−/−^and a cooperative increase could be observed in *mutyh*^−/−^ *ogg1*^−/−^, suggesting a cooperative function between OGG1 and MUTYH to prevent 8-oxoG-related mutagenesis. Russo et al. also reported an additive effect in *mutyh*^−/−^ *ogg1*^−/−^ on the age-dependent increase in 8-oxo-G levels in lung and small intestine compared to the single ko's (Russo et al., 2004). Strikingly, these tissues were identical with the ones that showed increased cancer incidence in *mutyh*^−/−^ *ogg1*^−/−^ mice in the study by Xie et al. (2004). MUTYH deficiency in a background of APC^min/+^ mice led to the occurrence of stop-codons in the APC gene by induction of CG→AT transversion mutations and thus promoted intestinal tumorigenesis (Sieber et al., 2004).

In 2007 a study reported an increased susceptibility to spontaneous and stress-induced tumorigenesis in a large cohort of *mutyh*^−/−^ mice kept for 18 months, strongly contradicting data on *mutyh*^−/−^ obtained by different groups thus far (Sakamoto et al., 2007). This suggested that presence of a MUTYH deficiency is sufficient to predispose for malignancies of the intestinal tract, such as lymphoma and adenoma. More impressively still, oral KBrO_3_ treatment of *mutyh*^−/−^ mice led to a dramatic increase in CG→AT transversion mutations and small intestinal tumors. The authors claimed that the tumor-prone phenotype might have been missed earlier due to genetic differences in the mouse strains and the older age at which the tumor burden was evaluated in their study. This was in line with the fact that many of the studies with *mutyh*^−/−^ mice have been reporting a strong tendency toward age-dependent accumulation of 8-oxo-G in tissues. In general, in light of the huge complexity of the disease, it can be debated, whether mice are useful cancer models to compare with the human disease, due to the entirely different life span, metabolism, inbreeding status and many other aspects.

As noted above, the combination *mutyh*^−/−^ and *msh2*^−/−^ did not greatly affect the mutation rate. However, the loss of *mutyh*^−/−^ combined with *msh2*^−/−^ significantly increased the amount of oxidative DNA damage in several organs compared to *msh2*^−/−^ mice, suggesting an independent contribution of both genes to genetic maintenance (Russo et al., 2009). Surprisingly, the development of metastasizing lymphoma and the time of death were significantly delayed in the *mutyh*^−/−^*msh2*^−/−^ mice compared to *msh2*^−/−^, suggesting that the cancer-prone phenotype of the double knockouts depends substantially on the activity of MUTYH (Russo et al., 2009). The relationship of MUTYH and MMR is reviewed in more detail in Russo et al. (2007).

In a mouse model of ulcerative colitis MUTYH was shown to play a major role in propagating the inflammatory response that lead to the onset of chronic colitis (Casorelli et al., 2010). Taken together, all the data analyzing the function of MUTYH *in vivo* strongly supports the idea that MUTYH-mediated correction of A:8-oxo-G mispairs plays an important role in the maintenance of genetic integrity and protects cells from malignant transformation.

## The MUTYH/Pol λ base-excision repair pathway

By catalyzing the excision of the mispaired A from A:8-oxo-G base pairs, MUTYH paves the way for a subsequent repair that ultimately reconstitutes an undamaged C:G base pair. MUTYH-initiated repair has been shown to involve a replication-coupled long-patch BER (LP-BER) pathway (Matsumoto, 2001; Parker et al., 2001; Yang et al., 2001; Parlanti et al., 2002). Along this line, a SP-BER pathway initiated by MUTYH was shown to be futile, because it uniquely generated A:8-oxo-G base pairs instead of the correct C:8-oxo-G base pairs, indicating that canonical MUTYH-initiated BER must proceed by the LP-BER sub-pathway (Hashimoto et al., 2004). For a long time it was unclear, which pol was capable of faithfully inserting a correct C opposite 8-oxo-G, as most examined pols showed significant error-prone bypass of 8-oxo-G (Shibutani et al., 1991; Pinz et al., 1995; Efrati et al., 1999; Prakash et al., 2000; Einolf and Guengerich, 2001; Vaisman and Woodgate, 2001; Krahn et al., 2003; Hsu et al., 2004). In 2007, our laboratory proposed that pol λ, together with its cofactors PCNA and replication protein A (RPA), inserts 1200-fold more efficiently the correct C opposite 8-oxo-G than the incorrect A (Maga et al., 2007). Furthermore, experiments with extracts from mouse embryonic fibroblasts (MEFs) deficient for pol λ suggested an important role of pol λ in bypass of 8-oxo-G. The importance of PCNA and RPA to determine the pol selection at 8-oxo-G lesions was further analyzed in a follow-up study. The two proteins were found to act as molecular switches to activate pol λ-dependent correct 8-oxo-G bypass and to repress the more error-prone pol β-dependent bypass (Maga et al., 2008). Subsequently, we showed that the MUTYH-initiated error-free LP-BER pathway involves pol λ (Maga et al., 2008; Van Loon and Hubscher, 2009), as depicted in detail in Figure 1. Herein, the monofunctional MUTYH excises the promutagenic A from A:8-oxo-G base pairs. This is followed by incision of the phosphodiester backbone 5′ to the AP site by APE 1 that generates a 3′OH and a 5′dRP moiety, respectively. Thereafter, in the presence of RPA and PCNA, pol λ incorporates the correct C opposite 8-oxo-G and further elongates the primer by one more nucleotide (nt) downstream, thus generating a short 1-nt 5′ flap. This overhang is processed by flap endonuclease 1 (Fen1), resulting in a product that can be ligated by DNA ligase I. The resulting C:8-oxo-G base pair is then substrate for the canonical OGG1-initiated SP-BER as discussed above.

## MUTYH-interacting proteins

All DNA damage repair pathways have to be tightly coordinated to ensure proper repair and to avoid the generation of cytotoxic and mutagenic intermediates. Protein-protein interactions either regulate the repair by recruitment of proteins to sites of DNA damage or modulate the catalytic activity of already bound enzymes.

MUTYH is interacting with proteins associated with the BER pathway, DNA replication and cell cycle checkpoints (Table 2). The first interaction partner of MUTYH was the endonuclease Ape1 (Parker et al., 2001; Yang et al., 2001). Ape1 stimulates the glycosylase activity of MUTYH independently from its own activity; a catalytically dead mutant of Ape1 still enhanced the cleavage efficiency of MUTYH on damaged DNA templates (Yang et al., 2001). Thus, the stabilization of the MUTYH-DNA complex was sufficient to enhance the repair capacity. Additionally, MUTYH and Ape1 were both recruited into a complex with A:8-oxo-G containing DNA in HeLa cell extracts (Gu and Lu, 2001). The interaction between the two proteins was suggested to be important to prevent the release of cytotoxic AP sites (Luncsford et al., 2010). MUTYH was found to interact with pol λ, as discussed above (Van Loon and Hubscher, 2009). Furthermore, the interaction of MUTYH with pol λ was enhanced by phosphorylation of pol λ by Cdk2/cyclinA (Markkanen et al., 2012b,c).

**Table 2:** **Interaction partners of MUTYH**.

**Interaction partner**	**Species**	**Interaction site in MUTYH**	**Stimulatory effect**
Ape1	*human*	259–318 (Parker et al., 2001)	Glycosylase activity (Yang et al., 2001)
MSH6	*human*	232–254 (Gu et al., 2002)	Glycosylase activity DNA binding (Gu et al., 2002)
Pol λ	*human*	Van Loon and Hubscher, 2009; Markkanen et al., 2012c 40–130 (Dorn et al., unpublished results)	n.d.
PCNA	*human*	505–527 (Parker et al., 2001), F518/F519 (Chang and Lu, 2002)	n.d.
	*S. pombe*	438–445 (Chang and Lu, 2002)	n.d.
9-1-1	*human*	295–350 (Shi et al., 2006) V315, E316 (Shi et al., 2006; Luncsford et al., 2010)	Glycosylase activity (Chang and Lu, 2005), interaction increased after IR (Shi et al., 2006)
	*S. pombe*	245–293 (Chang and Lu, 2005) I261, E262	Glycosylase activity (Chang and Lu, 2005), interaction increased after H_2_O_2_ treatment
RPA	*human*	6–32 (Parker et al., 2001)	n.d.
ATR	*human*	n.d.	Checkpoint mediator? (Hahm et al., 2011)

n.d., not determined.

Gu et al identified the mismatch repair protein MSH6 as further interaction partner of MUTYH, and MSH6 regulated MUTYH by stimulating its glycosylase activity and binding capacity to A:8-oxo-G containing DNA (Gu et al., 2002).

MUTYH interacts with PCNA and RPA under conditions of unperturbed DNA replication. It was suggested that, upon encountering DNA damage, MUTYH switches to interact with the heterotrimeric ring-like molecule Rad 9, Rad1, and Hus 1, called the 9-1-1 complex (Parker et al., 2001; Shi et al., 2006). Consistent with these findings, MUTYH co-localized with PCNA at replication foci in untreated cells (Boldogh et al., 2001). Also, replication was a prerequisite for MUTYH mediated repair to occur (Hayashi et al., 2002). The interaction site with PCNA was mapped to a conserved region within the MutY family, reflecting the importance of this interaction since PCNA directs MUTYH to the daughter strand where it excises a recently inserted mispaired A from A:8-oxo-G base pairs (Slupska et al., 1999). This directionality could also be the mechanism to make sure that MUTYH does not excise erroneously A from a base pair where 8-oxo-dGTP has been inserted opposite a templating (and thus correct) A. The interaction of MUTYH with PCNA and the structurally-related 9-1-1 complex was also confirmed in *S. pombe* (Parker et al., 2001; Chang and Lu, 2002, 2005; Shi et al., 2006; Luncsford et al., 2010). Interestingly, it was shown that even if the SpMUTYH does not have a perfect PCNA binding motif (Chang and Lu, 2005), cross-binding between the yeast and the human isoforms is possible and mutations within the PCNA binding domain impair the capability of MUTYH to repair A:8-oxo-G mismatches in yeast (Chang and Lu, 2002).

The 9-1-1 complex acts as a DNA damage sensor, blocks the cell cycle and simultaneously stimulates BER to allow repair to be completed before the DNA is replicated. The human MUTYH interacts with the h9-1-1 complex via binding to hRad1 and hHus1, but not to hRad9 (Shi et al., 2006). The glycosylase activity of MUTYH was stimulated by this interaction if 9-1-1 was present in a substantial molar excess. Treatment of cells with H_2_O_2_ or ionizing irradiation enhanced this interaction, supporting the hypothesis that 9-1-1 replaces PCNA in stress situations (Shi et al., 2006). Luncsford et al. identified the interdomain connector (IDC) of MUTYH to mediate the binding to 9-1-1 by providing a stabilized docking interface and proved the importance of the interaction by showing that mutations within this site decrease the repair of oxidative damage *in vivo* (Luncsford et al., 2010).

Partial interchangeability was observed between human and *S. pombe* homologs of these proteins, and enhanced glycosylase activity of *S. pombe* MUTYH was found with human Hus1 and the *S. pombe* 9-1-1. Human MUTYH was also observed to co-localize with Rad9 in cells treated with H_2_O_2_, suggesting that BER by MUTYH could be modulated by 9-1-1. Further work in *S. pombe* showed a decrease in repair of oxidative DNA damage *in vivo* when the interaction of MUTYH with 9-1-1 was disrupted, suggesting that this interplay significantly contributes to the response to oxidative stress (Luncsford et al., 2010). Also, MUTYH could be co-immunoprecipitated with ATR from human cells, possibly implicating MUTYH in ATR-mediated checkpoint execution (Hahm et al., 2011).

MUTYH from *S. pombe* was found to interact with Hst4, a histone deacetylase involved in silencing of genes and maintenance of genomic integrity, which seemed to regulate the levels of Hst4 after oxidative stress (Chang et al., 2011). Hst4 was further shown to interact also with the 9-1-1 complex. The association of MUTYH with telomeres was increased after oxidative stress and by deletion of Hst4, and Hst4 bound to telomeres decreased after oxidative stress, concomitant with a decrease in total Hst4 levels. Finally, MUTYH association with telomeres was increased in a Hst4 deletion background in the presence of chronic DNA damage caused by the lack of Hst4. Therefore, MUTYH seemed to regulate repair of telomeres by orchestrating the functions of 9-1-1 and Hst4. Finally, the WRN helicase/exonuclease was recently shown to promote MUTYH-initiated LP-BER of A:8-oxo-G mismatches by pol λ (Kanagaraj et al., 2012).

## Regulation of MUTYH

### Regulation of MUTYH levels

So far, only a limited amount of studies has been performed concerning the regulation of MUTYH levels. Respiratory hypoxia caused a strong increase in mtDNA damage and also in expression of MUTYH mRNA in rat brain (Englander et al., 2002). This suggested that the increase denoted an adaptive mechanism for protection of neuronal DNA from oxidative injuries stemming from an imbalance in metabolism. Follow-up work by the same group identified specific MUTYH isoforms exclusive to brain tissue in rats, that were targeted to the mitochondria and some of them were inducible upon respiratory hypoxia (Englander et al., 2002). The divergence in the N-terminus between the different MUTYH isoforms was found to influence their excision rates and the processing of AP sites (Ma et al., 2004). In mononuclear blood cells MUTYH levels were neither altered by hypoxia nor by inhalation of 10% oxygen for 2 h and the subsequent reoxygenation period in healthy human subjects, even though DNA strand breaks and oxidatively damaged purines accumulated by this treatment (Risom et al., 2007). MUTYH, together with SMUG1, was regulated transcriptionally by p73, a member of the p53 protein family, through DNA damage induction by bile acid exposure, suggesting that this interplay regulates DNA damage repair (Zaika et al., 2011).

A comparison of embryonic stem cells to more differentiated cells did not reveal any impact on the mRNA levels of MUTYH, in contrast to OGG1, which decreased upon differentiation (Kuboyama et al., 2011). Alimentary supplementation with quercetin, a plant-derived flavonoid that has been attributed with anticarcinogen, was found to enhance the expression of MUTYH in the distal colon mucosa of rats (Dihal et al., 2008). And finally, overexpression of hepatitis B virus X (HBx) was shown to increase 8-oxo-G levels in HepG2 cells, and to decrease the transcript levels of MUTYHα mRNA, while not affecting mRNA of OGG1, suggesting that this may be linked to the development of hepatocellular carcinoma which is associated to HBx infection (Cheng et al., 2009).

### Regulation of MUTYH by posttranslational modifications

Very little is known about the regulation of MUTYH by posttranslational modifications (PTM) (Table 3). Findings from Gu et al. showed that MUTYH could be phosphorylated *in vitro* by different protein kinases (Gu and Lu, 2001). Comparison of the activity of native MUTYH from human cell extracts with in recombinant MUTYH purified from bacteria revealed a dramatic difference in the glycosylase activity, probably due to the phosphorylation state of the proteins. Indeed, the dephosphorylation of native MUTYH led to a tremendous loss of function on A:G or A:8-oxo-G mismatch containing templates. Differences in activity were also described for recombinant MUTYH expressed in bacteria or insect cells (Kundu et al., 2010). Mass spectrometric analysis confirmed S524 to be phosphorylated in the more active MUTYH, expressed in insect cells. Further functional studies using wt, phosphomimetic, or phosphodeficient mutants revealed an important role of S524 in substrate recognition and binding to DNA.

**Table 3:** **Posttranslational modifications of MUTYH**.

**Posttranslational**	**Site of**	**Kinase**	**Stimulatory**
**modification**	**modification**		**effect**
Phosphorylation (Gu and Lu, 2001)	n.d.	n.d.	Glycosylase activity
Phosphorylation (Parker et al., 2002, 2003)	n.d.	PKC	Glycosylase activity
		PKA	
		Casein Kinase II	
Phosphorylation (Kundu et al., 2010)	S524	n.d.	DNA-binding

n.d., not determined.

A defect in phosphorylation of MUTYH was also found to cause a mutator phenotype in different microsatellite stable colorectal cancer cell lines (Parker et al., 2002). All tested cell lines that showed elevated 8-oxo-G levels showed a decline in repair of A:8-oxo-G mismatches. While the sequencing of the *MUTYH* locus in these cells did not reveal any mutations, the mRNA and protein levels of MUTYH were decreased. In a subsequent study the same authors could demonstrate that a loss of MUTYH phosphorylation by PKC was responsible for the observed increase in 8-oxo-G causing the mutator phenotype (Parker et al., 2003). The 8-oxo-G repair capacity in MUTYH impaired cell extracts could be restored by complementation with PKC, PKA or casein kinase II. Furthermore, the same effect could be achieved by treatment with the PKC activator phorbol-12-myristate-13-acetate (PMA). In contrast to that, no effect in cell extracts from MUTYH proficient cells occurred, indicating that MUTYH was already phosphorylated at a basal level in these cell lines. Consistent with these findings, MUTYH was a substrate for PKC *in vitro*. Finally, MUTYH purified directly from cell extracts treated with PMA showed an elevated capacity in the repair of A:8-oxo-G mismatches. So far it has not been elucidated whether phosphorylation only interferes with the catalytic activity of MUTYH, regulates its interaction with other proteins, or leads to a different subcellular localization. Since PKC can be stimulated by oxidative stress (Klein et al., 2000), it is possible that the phosphorylation-mediated regulation of MUTYH presents an adaptive response to DNA damage.

Taken together, it would be very interesting to investigate the regulation of MUTYH in more detail to get a better understanding how the different players of the 8-oxo-G repair machinery are controlled to protect cells from oxidative stress of endogenous or exogenous sources.

## Involvement of MUTYH in disease

### MAP (MUTYH associated polyposis)

Familial adenomatous polyposis (FAP) is an autosomal dominant disease characterized by the formation of hundreds to thousands of adenomatous polyps in the colons and rectums of the affected patients (reviewed in Fearnhead et al., 2001). It is caused by a germline mutation in the adenomatous polyposis coli (*APC)* gene, mutations that are also responsible for 80% of the sporadic colorectal tumors. In 2002, Al-Tassan and co-workers studied a British family with multiple colorectal adenoma and carcinoma, but failed to detect a mutation in the *APC* gene (Al-Tassan et al., 2002). Closer analysis of the patient material revealed an increased tendency of somatic CG→AT transversion mutations in the *APC* gene, which is consistent with 8-oxo-G mediated mutagenesis. This observation led the authors to dissect the three enzymes that work synergistically to counteract 8-oxo-G mediated mutagenesis, namely OGG1, MUTYH, and MTH. Sequencing of the respective genes revealed two non-conservative mutations in the *MUTYH* gene, Y165C (through an A→G transition) and G382D (through a G→A transition), while no pathogenic changes were observed in the *OGG1* and *MTH* genes. The two mutations were found to reduce the activity of *E. coli* mutY to remove A from G:A mismatches by 98% and 86%, respectively, suggesting that a defect in MUTYH activity was the reason for the accumulated mutations in the patients and thus responsible for the APC-like phenotype. Subsequent work revealed that these mutations not only compromise the bacterial mutY, but also caused a decrease in the activity of human MUTYH for excision of A opposite 8-oxo-G, which nicely correlated with the tumor phenotype (Al-Tassan et al., 2002; Chmiel et al., 2003; Pope and David, 2005). Further investigation led to the identification of seven other unrelated patients with colorectal adenomas or carcinomas that showed a bias of CG→AT transversion mutations to be carriers of biallelic germline mutations for MUTYH (Jones et al., 2002). This disorder is the only colorectal cancer form inherited in an autosomal recessive mode and is now commonly referred to as MAP, or infrequently also as FAP2 (http://www.omim.org). The prevalence of MAP is estimated to be around 1% of all colorectal cancer cases (Enholm et al., 2003; Croitoru et al., 2004; Fleischmann et al., 2004; Wang et al., 2004; Peterlongo et al., 2005; Webb et al., 2006; Kury et al., 2007; Cleary et al., 2009) and MUTYH mutations have been found in 7% (Filipe et al., 2009), and 10% (Pezzi et al., 2009) of FAP patients and 40% of AFAP patients, respectively (Filipe et al., 2009). The lifetime-cancer risk is assessed to 80% for colon cancer and 4% for duodenal cancer (Jasperson et al., 2010). Even though MAP is a rather recently discovered disease, many germline mutations in addition to the two found by Al-Tassan et al have been described so far. This is reflected in the abundance of literature investigating different single-nucleotide polymorphisms and their relevance to cancer development has been thoroughly reviewed in Cheadle and Sampson (2007) and Poulsen and Bisgaard (2008). Interestingly, other than MUTYH, no association of further genes involved in BER or the repair of oxidative DNA damage with a multiple colorectal adenoma phenotype has been found so far (Dallosso et al., 2008). Interestingly though, work by the Sweasy group has found that the *POLB* gene is mutated in many colorectal cancers, suggesting that at least some of these mutations may lead to compromised BER function in the affected tissues (Donigan et al., 2012; Nemec et al., 2012). MAP patients have been reported to be more prone also to extraintestinal tumors such as ovarian, bladder, skin, and breast cancer. For an overview of all extracolonic manifestations of MAP-patients, please refer to this recent review (Nielsen et al., 2011). For further clinical features, there are excellent recent reviews available (Jasperson et al., 2010; Nielsen et al., 2011). Several mutations in MUTYH associated with MAP were found to significantly enhance the spontaneous mutator phenotype of patient's lymphoblasts under conditions of oxidative stress and to accumulate 8-oxo-G in the DNA, underlining the role of MUTYH in the pathogenesis of this disease (Ruggieri et al., 2012). However, for many of the mutants it is unclear how the mutation affects its activity, and more work is needed to clarify their exact contribution to the disease.

### Equine cerebellar abiotrophy

Interestingly, MUTYH has been suggested to be involved in the pathogenesis of equine cerebellar abiotrophy, a neurological disease found in Arabian horses, as indicated by a SNP in the GATA2 binding region of the MUTYH promoter (Brault et al., 2011). Whether there is a real causative role and what mechanisms are behind it, remains to be elucidated by further studies.

## Conclusions and perspectives

The MUTYH DNA glycosylase is a remarkable enzyme since it has the specificity to remove an undamaged DNA base from a mismatch such as an A:8-oxo-G. It is found throughout evolution from bacteria to human, suggesting an essential role in preventing mutations arising from oxidative damage to the DNA. During the last three decades, our knowledge about how MUTYH functions has grown substantially. We now understand quite in detail how MUTYH acts catalytically, and the structures of prokaryotic and eukaryotic enzymes have been identified. However, the functional details of the at least 10 isoforms of MUTYH, are far from being unequivocally clarified. MUTYH acts together with pol λ in the so-called MUTYH/pol λ pathway that can replace a promutagenic A paired to an 8-oxo-G with a correct C. The interaction with the moving platforms PCNA and the 9-1-1 complex is apparently very important for the proper spatial and temporal engagement of MUTYH on the DNA, and there especially in the context of chromatin. So far, very little is known about the regulation of MUTYH, which is at least in part likely achieved by PTM. Phosphorylation as an important PTM contributes to regulate the activity of MUTYH. It is likely that other PTM's, such as ubiquitination, will be identified that govern the temporal (i.e., during the cell cycle) as well as the spatial (i.e., the subcellular localization) distribution of MUTYH. Also, the fact that mutations in MUTYH are identified in diseases of human and animals shifts this enzyme more and more into the focus of translational medicine. In the future, it will be of interest to understand more about the subcellular localization and specific functions of the different isoforms of MUTYH. Also, the exact regulation of the activity, stability, and localization of this enzyme is likely to yield many novel insights. Finally, we are anticipating further clarification of the functional roles of the different mutations in MUTYH associated with MAP.

### Conflict of interest statement

The authors declare that the research was conducted in the absence of any commercial or financial relationships that could be construed as a potential conflict of interest.
